# Early Postoperative Outcomes of the Direct Superior Approach versus the Posterior Approach in Total Hip Arthroplasty: A Systematic Review and Meta-Analysis

**DOI:** 10.3390/jcm13216291

**Published:** 2024-10-22

**Authors:** Kyun-Ho Shin, Jin-Uk Kim, Il-Tae Jang

**Affiliations:** 1 Department of Orthopedic Surgery, Juan Nanoori Hospital, Incheon 22147, Republic of Korea; 2 Department of Orthopedic Surgery, Incheon Nanoori Hospital, Incheon 21353, Republic of Korea; ultralex@mfnanoori.co.kr; 3 Nanoori Medical Research Institute, Seoul 06048, Republic of Korea; nanoori_research@naver.com

**Keywords:** direct superior approach, posterior approach, total hip arthroplasty, systematic review, meta-analysis

## Abstract

Background/Objectives: The direct superior approach (DSA) is a tissue-sparing alternative to the traditional posterior approach (PA) in total hip arthroplasty (THA), potentially offering improved recovery and fewer complications. This study compares perioperative parameters, radiological and functional outcomes, and complications between the DSA and the PA in primary THA. Methods: A systematic review and meta-analysis were conducted following PRISMA guidelines. Databases searched included MEDLINE/PubMed, Cochrane Library, Embase, and Scopus. Studies comparing the DSA and the PA in primary THA were included based on predefined criteria. Data extraction and quality assessment were performed independently by two authors. Statistical analyses included calculating standardized mean differences (SMD), odds ratios (OR), and 95% confidence intervals (CI). Heterogeneity was assessed using the χ2 test, I2 statistics, and sensitivity analyses. Results: Out of 126 identified articles, 10 studies were included, which encompassed 28,063 patients (DSA: 1464; PA: 26,599). Significant advantages of the DSA over the PA were observed in blood loss (SMD −0.26, *p* < 0.01), transfusion rate (OR 0.59, *p* = 0.03), length of stay (SMD −0.59, *p* < 0.01), discharge to home rates (OR 2.32, *p* < 0.01), and incision length (SMD −2.75, *p* < 0.01). No significant differences were found in radiological outcomes or most functional scores, although the DSA showed higher Harris Hip Scores at 1 month (SMD 0.77, *p* < 0.01). Conclusions: The DSA offers significant perioperative advantages over the PA, including reduced blood loss, transfusion rates, LOS, incision length, and improved early functional recovery with higher discharge to home rates. Comparable complication rates and radiological outcomes support the DSA’s safety and efficacy for quicker recovery in THA.

## 1. Introduction

Total hip arthroplasty (THA) is a well-established procedure known for its longevity and effectiveness in alleviating pain and improving function in patients with severe hip osteoarthritis and other debilitating hip conditions [1,2]. Over the past decade, there has been a growing emphasis on improving surgical methods for the hip joint to reduce hospital stays and promote faster recovery [3,4]. The choice of surgical approach for THA is primarily determined by the surgeon’s experience, training, and personal preferences [5]. Historically, the posterior approach (PA) has been the preferred method due to its extensive exposure and straightforward access, making it the most common technique used for THA. However, a recent trend has favored the direct anterior approach (DAA), known for its benefits of reduced dislocation revision rates, quicker mobilization, and shorter hospital stays [6,7,8,9]. Despite these advantages, the DAA is associated with a higher risk of femoral-sided revisions and requires a steep learning curve [7,10,11,12].

On the other hand, the direct superior approach (DSA) was introduced recently as another tissue-sparing alternative approach that aims to reduce soft tissue damage by preserving the iliotibial band and minimizing trauma to the short external rotators [13,14,15]. Since the DSA is a modification of the PA, it offers a familiar anatomical landscape for surgeons experienced with the posterior approach, resulting in a minimal learning curve [16,17].

Despite the growing interest and potential benefits of the DSA for THA, there are inconsistencies in the reported outcomes. Some studies suggest that the DSA leads to earlier functional recovery, lower blood loss, shorter hospital stays, and higher rates of home discharges compared to the PA [18,19]. Conversely, other studies report minimal differences in residual pain and clinical outcomes between the DSA and PA, indicating that the advantages of the DSA might be modest [20,21]. Previous systematic reviews on this topic have been inconclusive, partly due to the limited number of included studies [22]. However, recent high-quality comparative studies, including registry-based studies, have been published, providing new insights into the benefits of the DSA for THA [23,24]. This study aims to conduct a meta-analysis and systematic review comparing perioperative parameters, radiological and functional outcomes, and complications between the DSA and the PA in primary THA. The findings from this study will offer robust evidence to guide clinicians in selecting the optimal surgical approach for THA, ultimately improving patient outcomes.

## 2. Materials and Methods

This study followed the guidelines provided by the Preferred Reporting Items for Systematic Reviews and Meta-Analyses (PRISMA) to ensure the accuracy and transparency of our reporting [25]. This study is registered with the ResearchRegistry, and the unique identifying number is reviewregistry1892. Two authors independently performed study screening and selection, quality assessment, data extraction, and result pooling. To maintain consistency and resolve discrepancies, a third independent author reviewed the data and reached a consensus. The inter-reviewer reliability was assessed using the kappa statistic (κ), with values ranging from 0.92 to 1.00, indicating a high level of agreement between the reviewers. This rigorous methodology enhances the reliability and validity of our study findings.

### 2.1. Search Strategy

A comprehensive search was conducted on 4 May 2024, to identify relevant articles comparing the outcomes of the direct superior approach (DSA) and the posterior approach (PA) in total hip arthroplasty (THA). Databases searched included MEDLINE/PubMed, Cochrane Library, Embase, and Scopus. The search strategy utilized a combination of keywords and Medical Subject Headings (MeSH) terms within the [Title/Abstract] field. Terms included “total hip arthroplasty”, “total hip replacement”, “Arthroplasty, Replacement, Hip” [MeSH term] combined with “Direct superior approach”, “DSA”, “direct superior”, “iliotibial”, and “transpiriformis”. The search was limited to English-language studies to ensure consistency, acknowledging that this may introduce some bias, although it is generally accepted that including only English-language studies does not significantly affect the overall conclusions [26]. Additionally, reference lists of selected articles were reviewed to identify any potentially overlooked studies.

### 2.2. Inclusion and Exclusion Criteria

#### 2.2.1. Inclusion Criteria

1. Participants: Patients who underwent primary THA.
2. Interventions: The intervention group (DSA group) received primary THA using the DSA.
3. Comparisons: The control group (PA group) underwent primary THA using the conventional PA.
4. Outcomes: Assessed outcomes included peri-operative parameters (operating time, estimated perioperative blood loss, transfusion rate, length of stay (LOS)), radiologic outcomes (cup inclination, femoral stem alignment, leg length discrepancy (LLD)), and functional outcomes (Oxford hip score (OHS), Harris hip score (HHS), EQ-5D score, visual analog scale (VAS) for pain).
5. Follow-up: Studies required a minimum clinical follow-up of 3 months.
6. Study design: Both randomized controlled trials (RCTs) and comparative studies were eligible.

#### 2.2.2. Exclusion Criteria

Studies not meeting the inclusion criteria were excluded, including non-comparative studies, case reports, animal studies, studies of hemiarthroplasties and revisional arthroplasties, and studies lacking relevant outcome measures.

### 2.3. Study Screening, Data Collection, Quality Assessment and Certainty of Evidence

During the initial screening, duplicate publications were removed. Two independent authors then screened all titles and abstracts. The full texts of potentially relevant articles were reviewed in the second screening stage to confirm eligibility. Data extraction was performed by two independent authors and included: (1) study characteristics (first author, publication year, country), (2) patient demographics (number of patients, sex, age, BMI), (3) details of complications (periprosthetic fractures, infections, dislocations, nerve palsy), (4) follow-up duration, and (5) outcomes of interest. The Robins-I tool [27] was used for controlled, non-randomized studies, while the Risk of Bias (RoB) 2 tool [28] was applied for randomized trials. Risk of bias was visually represented using *robvis* [29]. Using the Grading of Recommendations, Assessment, Development, and Evaluation (GRADE) methodology, two independent authors assessed the degree of certainty of the evidence. Certainty of evidence could be high, moderate, low, or very low [30].

### 2.4. Statistical Analysis

Continuous outcomes were analyzed by calculating the standardized mean difference (SMD) and 95% confidence interval (CI). Dichotomous outcomes were analyzed using the odds ratio (OR) with a 95% CI. Meta-analyses were conducted to combine effects and calculate corresponding 95% CIs. Heterogeneity was assessed using the χ2 test, I^2^ statistics, and Q statistics. For I^2^ values less than 50%, a fixed-effect model (Mantel-Haenszel method) was used. For I^2^ values of 50% or higher, indicating significant heterogeneity, a “leave-one-out” sensitivity analysis was conducted to identify sources of heterogeneity. If heterogeneity persisted, a random-effects model (DerSimonian-Laird method) was applied [31]. Publication bias was assessed using Egger’s regression symmetry test [32]. All statistical analyses were conducted using Rstudio v.1.0.143 (RStudio Inc., Boston, MA, USA). Statistical significance was considered when *p* ≤ 0.05. Descriptive statistics were used for perioperative complications unsuitable for pooling, such as intra-operative and post-operative periprosthetic fractures, infections, dislocations, nerve palsy, and venous thromboembolism.

## 3. Results

### 3.1. Search Results

**Figure 1:**
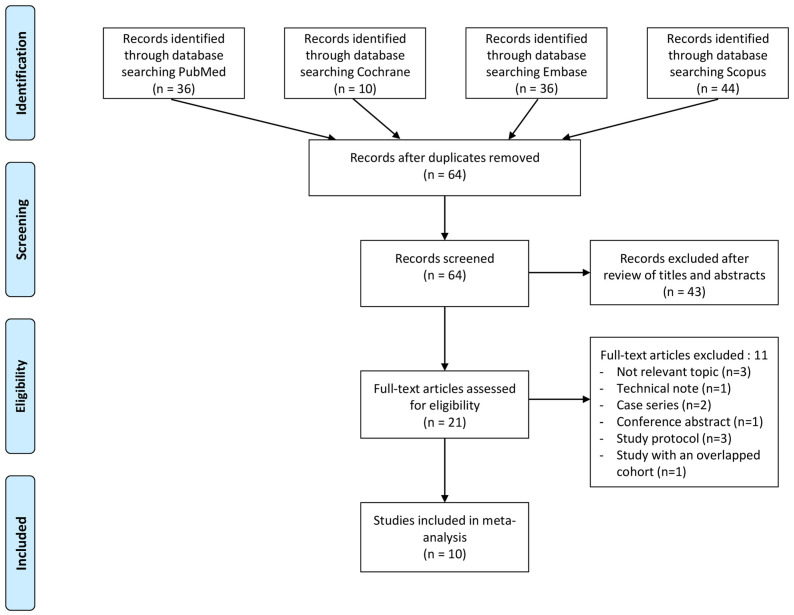
PRISMA flow diagram.

Figure 1 outlines the process used to identify and select studies for inclusion. Initially, a total of 126 articles were identified through the literature search. After removing 62 duplicates, the remaining 64 articles underwent screening based on their titles and abstracts. Subsequently, 21 full-text articles were assessed for eligibility. Eleven articles were excluded for not meeting the predetermined inclusion criteria. Ultimately, 10 articles were included in the meta-analysis [16,17,18,19,20,21,23,24,33,34].

### 3.2. Study Characteristics

Among the included studies, two were randomized controlled trials [19,34], two were prospective comparative studies [16,21], and six were retrospective comparative studies [17,18,20,23,24,33]. Table 1 presents the baseline characteristics of these studies. In total, 28,063 patients were included, comprising 1464 patients who underwent primary THA via the DSA and 26,599 patients who underwent primary THA via PA. The mean age of the patients ranged from 49.9 to 73.9 years, with follow-up periods ranging from 3 to 12 months.

### 3.3. Quality Assessment

**Figure 2:**
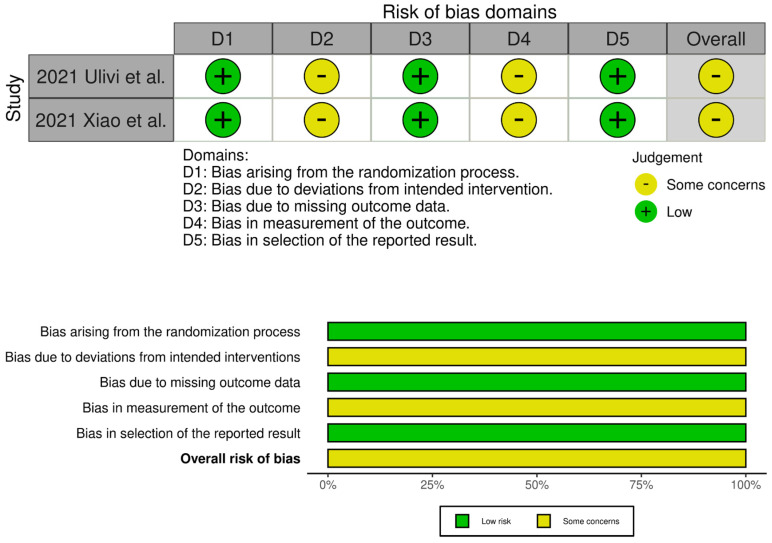
Traffic light plots of the quality assessment and summary plots of the distribution of risk-of-bias judgments in each bias domain of all outcomes assessed in studies judged with RoB 2 [19,34].

**Figure 3:**
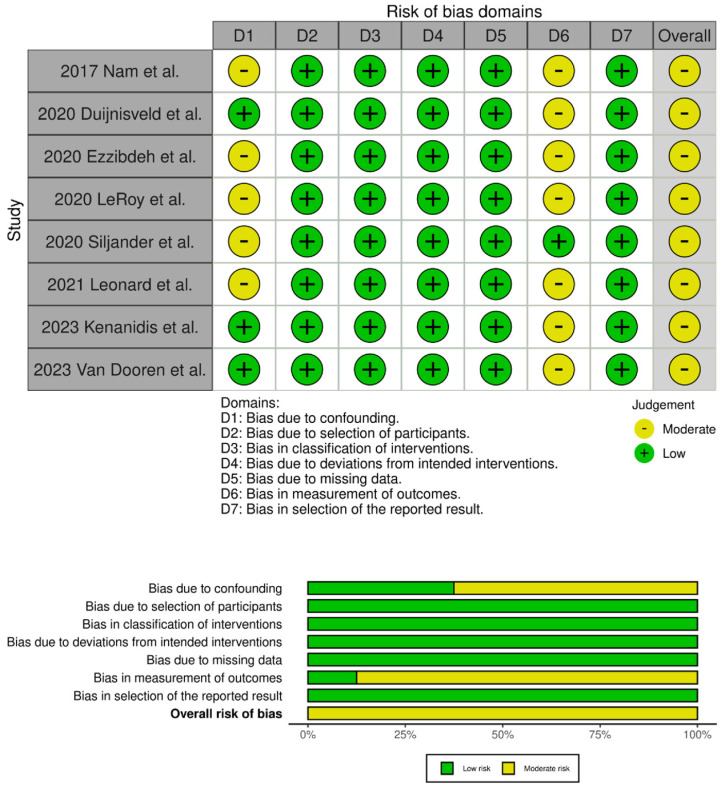
Traffic light plots of the quality assessment and summary plot of the distribution of risk-of-bias judgments in each bias domain of all outcomes assessed in studies judged with Robins–I [16,17,18,20,21,23,24,33].

The evaluation of the risk of bias in the included studies is depicted in Figure 2 and Figure 3. Two RCTs were assessed to have an unclear risk of performance and detection bias [19,34]. Overall, these studies were categorized as having some concerns of bias. Among the eight comparative studies, seven exhibited a risk of bias in the measurement of outcomes [16,17,20,21,23,24,33], and five exhibited a risk of bias due to confounding [17,18,20,21,33]. Overall, all studies were categorized as having some concerns of bias.

### 3.4. Meta-Analysis Results

#### 3.4.1. Perioperative Parameters

The findings of the meta-analyses regarding perioperative parameters are summarized in Table 2. Significant intergroup differences in favor of the DSA group were observed for estimated perioperative blood loss (SMD −0.26, 95% CI: −0.43 to −0.09, *p* < 0.01, I^2^ = 43%), transfusion rate (OR 0.59, 95% CI: 0.36 to 0.94, *p* = 0.03, I^2^ = 0%), LOS (SMD −0.59, 95% CI: −0.77 to −0.41, *p* < 0.01, I^2^ = 69%), rate of discharge to home (OR 2.32, 95% CI: 1.75 to 3.07, *p* < 0.01, I^2^ = 15%), and incision length (SMD −2.75, 95% CI: −3.21 to −2.30, *p* < 0.01, I^2^ = 0%).

#### 3.4.2. Radiological and Functional Outcomes and Complications

The radiological and functional outcomes are summarized in Table 2. A significant difference was found for LLD (SMD 0.29, 95% CI: 0.07 to 0.52, *p* = 0.01, I^2^ = 0%). However, there were no significant differences in cup inclination angle or femoral stem alignment. For functional outcomes, the DSA group showed a significant advantage in HHS at 1 month postoperatively (SMD 0.77, 95% CI: 0.24 to 1.30, *p* < 0.01, I^2^ = 79%). There were no significant differences in postoperative VAS scores during activity and rest at 3 months, or in EQ-5D score, HHS, and OHS at 3 and 12 months. Perioperative complications are summarized in Table 3, showing no significant differences between the DSA and the in terms of dislocations, infections, fractures, and thromboembolic events.

#### 3.4.3. Sensitivity Analyses

Significant heterogeneity was observed in the pooled results for operating time, LOS, cup inclination angle, incision length, and postoperative 1-month HHS. Sensitivity analyses identified the study by Xiao et al. as a potential source of heterogeneity for incision length and LOS [34]. Consequently, this study was excluded from the meta-analyses, as shown in  Supplementary Figures S1–S4. However, substantial heterogeneity remained in the LOS results. The study by Leonard et al. was identified as a source of heterogeneity for the cup inclination angle [20], leading to its exclusion from the meta-analyses, as shown in Supplementary Figures S5 and S6.

## 4. Discussion

This systematic review and meta-analysis compared the early postoperative outcomes of the DSA and the PA in primary THA. The findings show significant advantages of the DSA in terms of perioperative blood loss, transfusion rate, LOS, discharge to home rates, and incision length. Radiological outcomes, such as cup inclination angle and femoral stem alignment, showed no significant differences between the approaches. Functional outcomes indicated a significant advantage for the DSA in HHS at 1 month, while no significant differences were observed in other functional measures at 3 and 12 months. Perioperative complications, including dislocations, infections, fractures, and thromboembolic events, were comparable between the two approaches.

Over a million total hip arthroplasty (THA) procedures are performed annually, and this number is expected to continue rising [1,35]. This surge significantly impacts healthcare budgets, leading to a greater emphasis on reducing hospital stays and increasing home discharges to manage the costs associated with THA [36]. Additionally, to counteract rising costs, some surgeons have started incorporating outpatient THA into their practice, allowing patients to be discharged on the same day as their surgery. The growing prevalence of THA procedures, especially in outpatient settings, has intensified the focus on achieving faster recovery and earlier functional improvements through minimally invasive surgical techniques [37,38]. The direct superior approach (DSA) was designed to reduce surgical trauma to the soft tissues surrounding the hip joint by preserving the iliotibial band, utilizing a modified capsulotomy, and minimizing muscle damage to the quadratus femoris and gluteus minimus [13,14,15].

A previous systematic review has been conducted to evaluate the efficacy of the DSA in THA. However, clinical evidence of the DSA in RCR remains unclear, and the reliability of the conclusions is questionable due to the limited number of studies included [22]. With growing interest in the DSA as a muscle and tissue-sparing approach for THA, recent high-quality comparative studies have been published [23,24]. Therefore, the purpose of this study was to identify, summarize, and synthesize the currently available clinical results on the DSA compared with the conventional PA in primary THA. The findings demonstrate that the DSA not only offers cosmetic advantages by reducing incision length but also decreases blood loss and transfusion rates, shortens LOS, and improves early functional recovery, as indicated by higher HHS at 1 month postoperatively. These results suggest that the DSA could be a better option than the PA, particularly for patients requiring earlier recovery.

The reported disadvantages of minimally invasive approaches include longer operating times, a smaller field of view leading to technical errors such as component malpositioning, and increased intraoperative complications, including periprosthetic fractures [7,39,40,41]. However, despite its minimally invasive nature and muscle-sparing advantages, the DSA did not result in significantly longer operating times compared to the PA. Unlike the steep learning curve associated with the DAA, the DSA is a modification of the familiar PA, resulting in a shorter learning curve, often requiring fewer than 20 cases to master [16,17]. Furthermore, studies have shown that the DSA causes less soft tissue damage compared to both DAA and PA, as evidenced by laboratory markers and cadaveric muscle and tendon assessments [34,42]. This reduced muscle damage likely accounts for the lower blood loss and decreased transfusion rates observed, which are clinically significant.

Additionally, by preserving the iliotibial band and short external rotators such as the quadratus femoris, the DSA facilitates early rehabilitation, leading to better early gait outcomes. Although this study did not perform a meta-analysis on gait parameters, previous research indicates that patients undergoing the DSA show superior Timed Up and Go test results compared to the PA at discharge and at 3 months postoperatively [17,19]. The ability to facilitate early ambulation and accelerated rehabilitation accounts for the reduced LOS and higher discharge to home rates observed in our review. While no significant differences were found in VAS, HHS, OHS, and EQ-5D scores at 3 and 12 months, the significant improvement in HHS at 1 month postoperatively highlights the DSA’s role in earlier functional recovery.

Radiological outcomes also showed no significant differences, except for a slight but clinically insignificant difference in LLD. The absence of significant differences in complications such as periprosthetic joint infection, dislocation, periprosthetic fracture, and nerve palsy further supports the DSA’s safety and reliability. Unlike the DAA, which has been associated with an increased risk of periprosthetic femur fractures, the DSA maintains a similar surgical field to the PA, minimizing such risks. Additionally, a recent population-based cohort study reported lower dislocation rates and revision risks with the DSA compared to the PA. Future studies should investigate whether the superior capsular incision and preservation of the quadratus femoris in the DSA can indeed reduce dislocation and revision risks [43].

The DSA, as a muscle- and tissue-sparing approach, offers several advantages without compromising the positioning of the acetabular and femoral prostheses. It supports rapid recovery of gait and hip function with comparable complication risks to the PA, making it a safe and reliable surgical option worthy of broader application. The DSA is particularly beneficial for younger patients or those undergoing outpatient THA, as well as very elderly patients with hip fractures, by reducing blood loss and transfusion rates and facilitating early ambulation and rehabilitation.

This study has several limitations. First, the inclusion of a limited number of studies, as well as the combination of RCTs and non-randomized comparative studies, may introduce potential biases. While RCTs are regarded as the gold standard for causal inference, incorporating observational studies can improve the precision and accuracy of the findings by increasing the overall sample size and data diversity [44]. This approach provides more comprehensive and reliable results, even though it may introduce some heterogeneity. Second, the included studies exhibited significant heterogeneity in preoperative diagnosis, outcome assessment methods, rehabilitation protocols, and follow-up periods. Additionally, the strategies to minimize blood loss were not consistently reported across studies. This variability should be taken into account when interpreting the findings, as it may influence the generalizability of the results. Third, despite rigorous sensitivity analyses, high heterogeneity in some parameters, such as operating time, LOS, and HHS, suggests that these results should be interpreted with caution. Lastly, the focus on short-term outcomes limits the ability to draw conclusions about long-term benefits. Future well-designed RCTs with larger sample sizes, longer follow-up periods, and better control of confounding factors are needed to provide more robust evidence.

## 5. Conclusions

In conclusion, this study provides strong evidence that the DSA offers significant perioperative advantages over the PA, including reduced blood loss, transfusion rates, LOS, and incision length, along with improved early functional recovery and higher discharge-to-home rates. The comparable complication rates and radiological outcomes further support the DSA’s safety and efficacy. These findings provide valuable insights and suggest that the DSA is a viable option for THA, especially for patients requiring quicker recovery and early rehabilitation.

## Figures and Tables

**Table 1:** Characteristics of included studies.

First Author (Year)	Study Design	Country	Level of Evidence	Sample Size (*n*)		Mean Age (Years)		Male *n* (%)		BMI (kg/m^2^)		Follow Up	
				DSA	PA	DSA	PA	DSA	PA	DSA	PA	DSA	PA
Nam et al. (2017) [21]	Prospective comparative study	USA	II	42	196	63.9	49.9	NR	NR	NR	NR	12 months	
Duijnisveld et al. (2020) [16]	Prospective comparative study	Netherlands	II	52	52	69	69	24 (46)	18 (35)	25	25	12 months	
Ezzibdeh et al. (2020) [17]	Retrospective comparative study	USA	III	20	20	51	64	10 (50)	13 (65)	26	29	Mean 3.1 months	Mean 3.0 months
LeRoy et al. (2020) [33]	Retrospective comparative study	USA	III	403	273	63.4	63.4	190 (47.1)	138 (50.5)	28.1	31.6	12 months	
Siljander et al. (2020) [18]	Retrospective comparative study	USA	III	333	3162	62	64	154 (46.2)	1363 (43.1)	28.8	30.2	3 months	
Leonard et al. (2021) [20]	Retrospective comparative study	UK	III	100	100	68	68.1	61 (61)	61 (61)	28.0	28.9	12 months	
Ulivi et al. (2021) [19]	Randomized controlled trial	Italy	II	22	23	74	72	7 (31.8)	10 (43.5)	23.1	23.8	6 months	
Xiao et al. (2021) [34]	Randomized controlled trial	China	II	49	57	71.1	73.9	16 (32.7)	26 (45.6)	26.7	26.4	3 months	
Kenanidis et al. (2023) [23]	Retrospective comparative study	Greece	III	100	100	65.4	65.5	42 (42)	37 (37)	28.4	27.9	12 months	
Van Dooren et al. (2023) [24]	Retrospective comparative study	Netherlands	III	343	22,616	<60; 57 (17%) 60–74; 199 (58%) ≥75; 87 (25%)	<60; 3277 (15%) 60–74; 12,973 (57%) ≥75; 6361 (28%)	120 (35)	8655 (38)	<18.5; 3 (1%) 18.5–25; 155 (45%) 25–30; 131 (38%) 30–40; 53 (16%) >40; 1 (0.3%)	<18.5; 122 (1%) 18.5–25; 6859 (30%) 25–30; 9811 (44%) 30–40; 5486 (24%) >40; 277 (1%)	12 months	

DSA, direct superior approach; PA, posterior approach; BMI, body mass index.

**Table 2:** Summary of perioperative parameters, radiological, and functional outcomes between the DSA and PA groups.

	OR or SMD	LL 95%CI	UL 95%CI	*p* Value	Heterogeneity (%)	Analysis Model	Egger’s Test (*p* Value)	Grading of Recommendation Assessment, Development, and Evaluation							
								Number of Studies	Study Design	Risk of Bias	Inconsist-ency	Indirect- ness	Impreci-sion	Other Consideration	Certainty of Evidence with Explanations for Downgrading of Evidence
Operating time	−0.1	−0.62	0.42	0.70	95	Random	0.72	7	RCTs and Non-RCTs	Not serious	Serious ^a^	Not serious	Not serious	None	Low ^a^
Estimated perioperative blood loss	−0.26	−0.43	−0.09	<0.01	43	Fixed	0.22	4	RCTs and Non-RCTs	Not serious	Not serious	Not serious	Not serious	None	Moderate
Hgb Drop	−0.08	−0.35	0.19	0.55	0	Fixed	NA	2	RCTs and Non-RCTs	Not serious	Not serious	Not serious	Serious ^b^	None	Low ^b^
Transfusion rate	0.59	0.36	0.94	0.03	0	Fixed	0.63	3	Non-RCTs	Not serious	Not serious	Not serious	Not serious	None	Moderate
Length of stay	−0.59	−0.77	−0.41	<0.01	69	Random	0.23	6	Non-RCTs	Not serious	Serious ^c^	Not serious	Not serious	None	Low ^c^
Discharge to home	2.32	1.75	3.07	<0.01	15	Fixed	0.44	5	Non-RCTs	Not serious	Not serious	Not serious	Not serious	None	Moderate
Cup inclination	−0.32	−0.65	0.01	0.06	43	Fixed	NA	2	Non-RCTs	Not serious	Not serious	Not serious	Serious ^d^	None	Low ^d^
Femoral stem alignment	0.06	−0.17	0.28	0.61	0	Fixed	NA	2	Non-RCTs	Not serious	Not serious	Not serious	Serious ^e^	None	Low ^e^
Leg length discrepancy	0.29	0.07	0.52	0.01	0	Fixed	NA	2	Non-RCTs	Not serious	Not serious	Not serious	Serious ^f^	None	Low ^f^
Incision length	−2.75	−3.21	−2.30	<0.01	0	Random	NA	2	RCTs and Non-RCTs	Not serious	Not serious	Not serious	Serious ^g^	None	Low ^g^
EQ–5D score at 3 months	0.01	−0.09	0.11	0.88	0	Fixed	NA	2	Non-RCTs	Not serious	Not serious	Not serious	Serious ^h^	None	Low ^h^
EQ–5D score at 12 months	0.00	−0.10	0.11	0.93	0	Fixed	NA	2	Non-RCTs	Not serious	Not serious	Not serious	Serious ^i^	None	Low ^i^
HHS at 1 month	0.77	0.24	1.30	<0.01	79	Random	NA	2	RCTs and Non-RCTs	Not serious	Serious ^j^	Not serious	Serious ^k^	None	Low ^j,k^
HHS at 3 months	0.17	−0.02	0.36	0.07	0	Fixed	0.10	4	RCTs and Non-RCTs	Not serious	Not serious	Not serious	Not serious	None	Moderate
HHS at 12 months	−0.09	−0.31	0.14	0.45	0	Fixed	NA	2	Non-RCTs	Not serious	Not serious	Not serious	Serious ^l^	None	Low ^l^
OHS at 3 months	0.01	−0.09	0.10	0.90	0	Fixed	0.70	3	Non-RCTs	Not serious	Not serious	Not serious	Not serious	None	Moderate
OHS at 12 months	0.01	−0.08	0.11	0.78	0	Fixed	0.49	3	Non-RCTs	Not serious	Not serious	Not serious	Not serious	None	Moderate
VAS during activity at 3 months	−0.01	−0.11	0.09	0.83	0	Fixed	NA	2	Non-RCTs	Not serious	Not serious	Not serious	Serious ^m^	None	Low ^m^
VAS during rest at 3 months	0.01	−0.10	0.11	0.89	42	Fixed	NA	2	Non-RCTs	Not serious	Not serious	Not serious	Serious ^n^	None	Low ^n^

DSA, direct superior approach; PA, posterior approach; OR, odds ratio; SMD, standardized mean difference; LL, lower limit; CI, confidence interval; UL, upper limit; Hgb, hemoglobin; NA, not applicable; EQ–5D, EuroQol-5 Dimension; HHS, Harris Hip Score; OHS, Oxford Hip Score; VAS, visual analog scale. ^a, c, j^ Statistical heterogeneity and inconsistency in direction of effect; ^b, d, e, f, g, h, I, k, l, m, n^. Two studies with small sample size and wide confidence interval.

**Table 3:** Summary of perioperative complications between the DSA and PA groups.

First Author (Year)	Complications	
	DSA	PA
Nam et al. (2017) [21]	Patients were excluded if they had a history of postoperative infection, fracture, dislocation, or revisional surgery.	
Duijnisveld et al. (2020) [16]	Two patients with postoperative Vancouver B2 periprosthetic femoral fractures requiring femoral stem revision occurred. One occurred after a traffic accident, and the other was regarded as surgery related because of no adequate trauma at 3 weeks follow up. No infection and thromboembolic events were observed.	One postoperative Vancouver B2 periprosthetic femoral fracture requiring femoral stem revision occurred without an adequate trauma 4 weeks postoperatively. Two dislocations, with one patient who received revision to a longer femoral head, and the other who received a closed reduction. No infection or thromboembolic events were observed. One ischial neuropathy occurred.
Ezzibdeh et al. (2020) [17]	No dislocation, periprosthetic fracture, periprosthetic joint infection, or other postoperative surgical complications were observed.	No dislocation, periprosthetic fracture, periprosthetic joint infection, or other postoperative surgical complications were observed.
LeRoy et al. (2020) [33]	No dislocations, wound infections, or neuropraxias were observed during follow-up.	No dislocations, wound infections, or neuropraxias were observed during follow-up.
Siljander et al. (2020) [18]	Readmission 4.5% ED visit 5.1% Intraoperative fracture 0.9% Postoperative fracture 1.2% Dislocation 0.6% DVT/PE 0.6% No periprosthetic joint infection	Readmission 4.9% ED visit 6.8% Intraoperative fracture 0.6% Postoperative fracture 0.6% Dislocation 0.8% DVT/PE 0.7% Periprosthetic joint infection 0.3%
Leonard et al. (2021) [20]	No intraoperative complications were observed. One postoperative Vancouver B2 periprosthetic femoral fracture requiring femoral stem revision occurred due to a syncopal episode.	No intraoperative complications were observed.
Ulivi et al. (2021) [19]	One periprosthetic fracture and two anterior dislocations due to falls. No other adverse events were observed.	One ischemic stroke occurred. No other adverse events were observed.
Xiao et al. (2021) [34]	NR	NR
Kenanidis et al. (2023) [23]	One dislocation requiring a closed reduction occurred 3 weeks postoperatively. One superficial wound infection occurred. No periprosthetic joint infections, sciatic nerve palsies, intraoperative fractures, or thromboembolic events were observed.	One superficial wound infection occurred. No periprosthetic joint infections, sciatic nerve palsies, intraoperative fractures, or thromboembolic events were observed.
Van Dooren et al. (2023) [24]	NR	NR

DSA, direct superior approach; PA, posterior approach; ED, emergency department; DVT/PE, deep vein thrombosis/pulmonary embolism; NR, not reported.

## Data Availability

The data presented in this study are available upon request from the corresponding author.

## Supplementary Materials

The following supporting information can be downloaded at: https://www.mdpi.com/article/10.3390/jcm13216291/s1: Figure S1: Incision length before sensitivity analyses, Figure S2: Sensitivity analysis for incision length, Figure S3: Length of stay before sensitivity analyses, Figure S4: Sensitivity analysis for length of stay, Figure S5: Cup inclination angle before sensitivity analyses, Figure S6: Sensitivity analysis for cup inclination angle.
