# Supplementary material for: Early Postoperative Outcomes of the Direct Superior Approach versus the Posterior Approach in Total Hip Arthroplasty: A Systematic Review and Meta-Analysis
Source: J Clin Med. 2024 Oct 22;13(21):6291. doi: 10.3390/jcm13216291 (PMC11546740; doi:10.3390/jcm13216291)
Supplement: Supplementary file 1 [file jcm-13-06291-s001.zip › jcm-3189385-supplementary.pdf]

Study	SMD	SE	Weight (common)	Weight (random)	SMD [95% CI]
Ezzibdeh et al. 2020	−2.4188	0.4161	24.8%	33.1%	−2.42 [−3.23; −1.60]
Xiao et al. 2021	−5.9389	0.4520	21.0%	32.7%	−5.94 [−6.82; −5.05]
Kenanidis et al. 2023	−2.9060	0.2812	54.2%	34.2%	−2.91 [−3.46; −2.35]
**Total (common effect, 95% CI)**			**100.0%**	**.**	**−3.42 [−3.83; −3.02]**
**Total (random effect, 95% CI)**			**.**	**100.0%**	**−3.74 [−5.66; −1.82]**

Heterogeneity: $Tau^2 = 2.7274$; $Chi^2 = 40.18$, df = 2 ($P < 0.01$); $I^2 = 95\%$
Test for overall effect (common effect): Z = −16.52 ($P < 0.01$)
Test for overall effect (random effects): Z = −3.82 ($P < 0.01$)

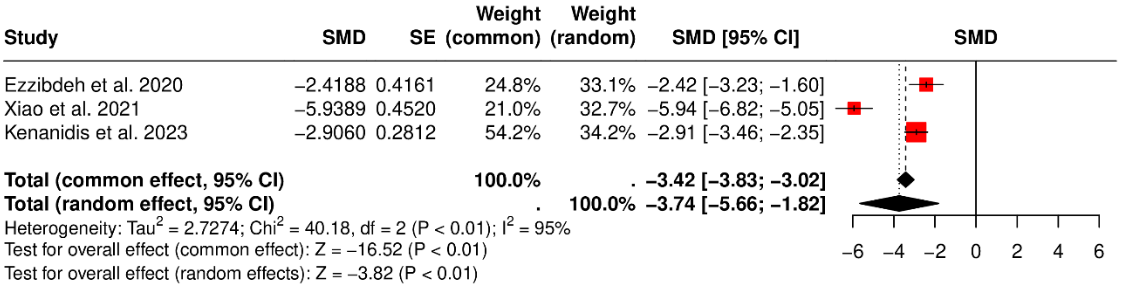

Supplementary Figure S1. Incision length before sensitivity analyses

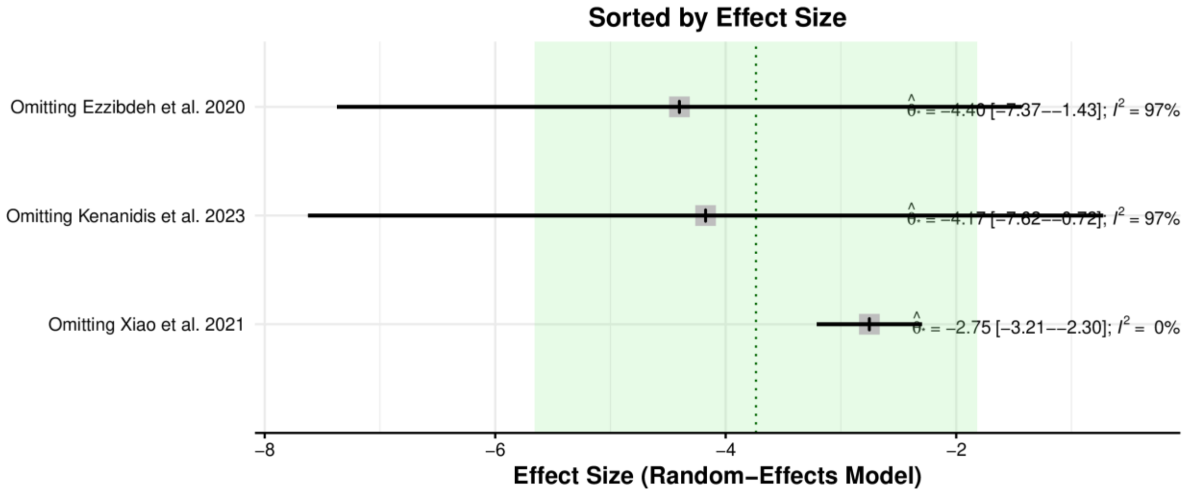

Supplementary Figure S2. Sensitivity analysis for incision length

Study	SMD	SE	Weight (common)	Weight (random)	SMD [95% CI]
Duijnisveld et al. 2020	−0.3475	0.1976	4.3%	13.9%	−0.35 [−0.73; 0.04]
Ezzibdeh et al. 2020	−1.0286	0.3365	1.5%	10.2%	−1.03 [−1.69; −0.37]
LeRoy et al. 2020	−0.5128	0.0796	26.4%	16.5%	−0.51 [−0.67; −0.36]
Siljander et al. 2020	−0.4410	0.0579	50.0%	16.8%	−0.44 [−0.55; −0.33]
Leonard et al. 2021	−0.5485	0.1441	8.1%	15.2%	−0.55 [−0.83; −0.27]
Xiao et al. 2021	−2.5067	0.2600	2.5%	12.2%	−2.51 [−3.02; −2.00]
Kenanidis et al. 2023	−1.0274	0.1505	7.4%	15.1%	−1.03 [−1.32; −0.73]
**Total (common effect, 95% CI)**			**100.0%**	**.**	**−0.57 [−0.65; −0.49]**
**Total (random effect, 95% CI)**			**.**	**100.0%**	**−0.86 [−1.19; −0.52]**

Heterogeneity: $Tau^2 = 0.1690$; $Chi^2 = 73.36$, df = 6 ($P < 0.01$); $I^2 = 92\%$
Test for overall effect (common effect): $Z = -13.88$ ($P < 0.01$)
Test for overall effect (random effects): $Z = -5.04$ ($P < 0.01$)

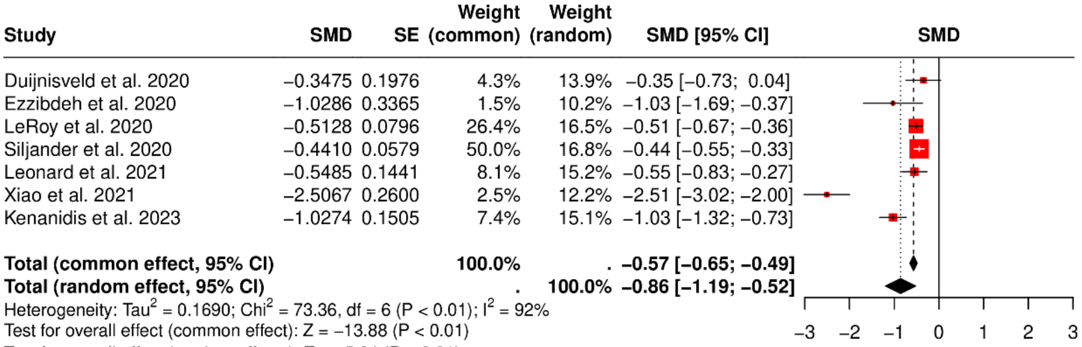

Supplementary Figure S3. Length of stay before sensitivity analyses

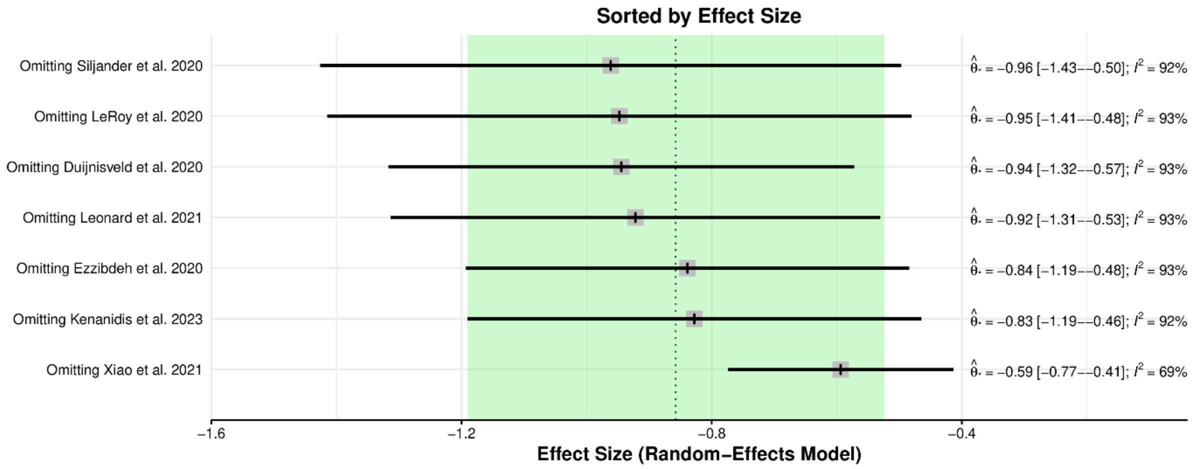

Supplementary Figure S4. Sensitivity analysis for length of stay

Study	SMD	SE	Weight (common)	Weight (random)	SMD [95% CI]
Duijnisveld et al. 2020	−0.4602	0.1987	30.5%	34.3%	−0.46 [−0.85; −0.07]
Ezzibdeh et al. 2020	0.0329	0.3162	12.0%	29.8%	0.03 [−0.59; 0.65]
Leonard et al. 2021	0.6048	0.1446	57.5%	35.9%	0.60 [0.32; 0.89]
**Total (common effect, 95% CI)**			**100.0%**	**.**	**0.21 [−0.00; 0.43]**
**Total (random effect, 95% CI)**			**.**	**100.0%**	**0.07 [−0.66; 0.80]**

Heterogeneity: $Tau^2 = 0.3669$; $Chi^2 = 19.14$, df = 2 ($P < 0.01$); $I^2 = 90\%$
Test for overall effect (common effect): $Z = 1.93$ ($P = 0.05$)
Test for overall effect (random effects): $Z = 0.19$ ($P = 0.85$)

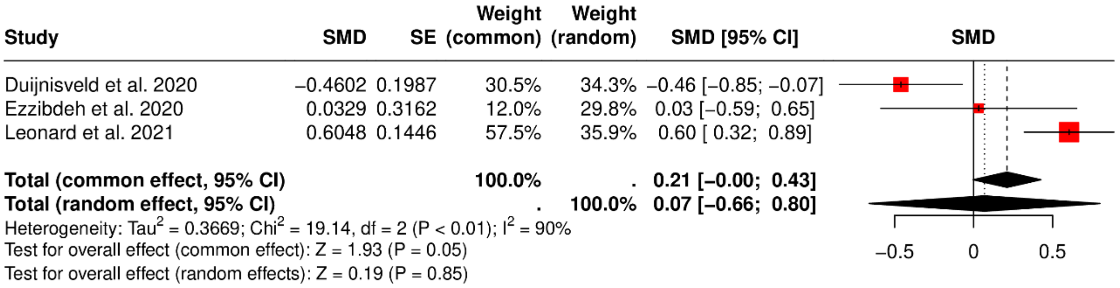

Supplementary Figure S5. Cup inclination angle before sensitivity analyses

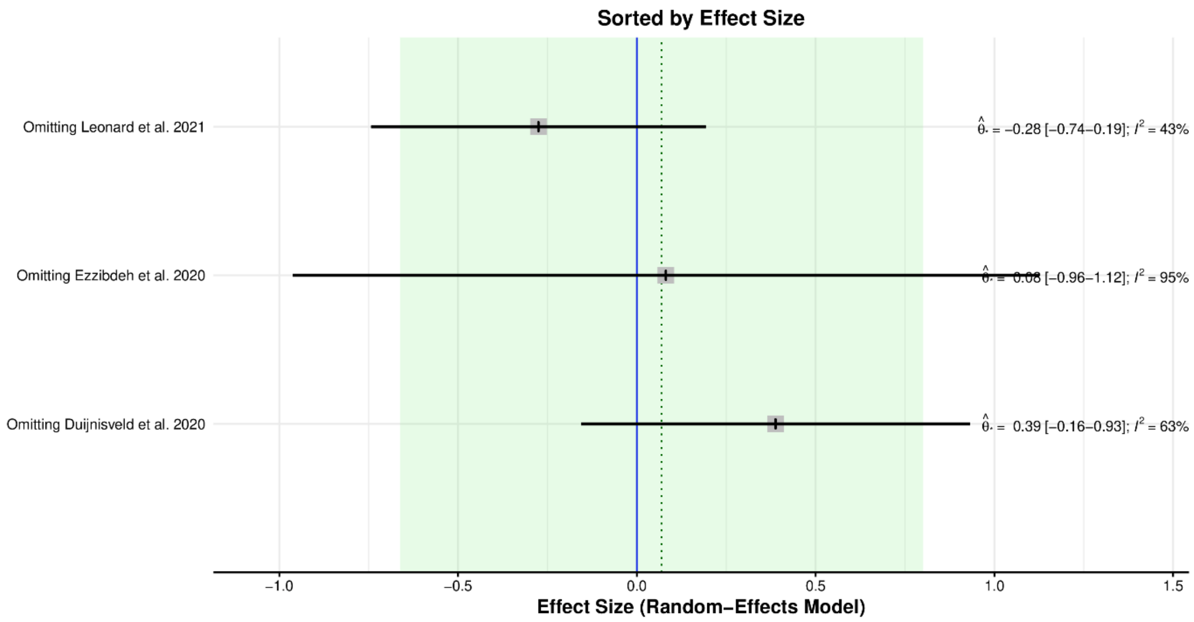

Supplementary Figure S6. Sensitivity analysis for Cup inclination angle
